# Complete recovery after complement factor I deficiency associated fulminant acute hemorrhagic leukoencephalitis: a case report

**DOI:** 10.3389/fimmu.2025.1586288

**Published:** 2025-06-18

**Authors:** Fanni Szumutku, Léna Szabó, Zoltán Liptai, Edit Varga, Tímea Seszták, Péter Barsi, Ádám Goschler, Gábor Szarvas, Klára Horváth, Simon Péter Nagy, Zoltán Prohászka, Ágnes Szilágyi, Sarolta Dobner

**Affiliations:** ^1^ Tűzoltó Street Department, Pediatric Center, Semmelweis University, Budapest, Hungary; ^2^ Department of Neuroradiology, Medical Imaging Center, Semmelweis University, Budapest, Hungary; ^3^ Research Laboratory, Department of Internal Medicine and Hematology, Semmelweis University, Budapest, Hungary

**Keywords:** acute hemorrhagic leukoencephalitis, complement factor I deficiency, neuroinflammatory disease, immune-mediated neurological disorder, case report

## Abstract

**Introduction:**

Acute hemorrhagic leukoencephalitis (AHLE) is a rare, fulminant neuroinflammatory disease with high mortality rate. It most often occurs after infections; however, the exact etiology of the disease remains unclear. We highlight that complement factor I (FI) deficiency may be a possible cause of AHLE.

**Case report:**

We describe a 9-year-old patient presenting with fever, headache, dizziness, ataxia, and diplopia, who developed rapid neurologic decline and refractory intracranial pressure elevation. Based on clinical, laboratory, and MRI findings, AHLE was diagnosed. Successful treatment included therapeutic plasma exchange (PEX) and early decompressive craniectomy. At one year of follow-up, the patient showed complete recovery. Complement testing of the patient revealed complete FI deficiency. Genetic workup uncovered a germline pathogenic variant in the *CFI* gene.

**Discussion:**

As AHLE is an emerging phenotype of complement FI deficiency, with only a few previously reported cases in the literature, high clinical suspicion and awareness among clinicians are needed. To control the complement system, prompt blockade with complement FI substitution via PEX and early decompressive craniectomy may be life-saving. In neuroinflammatory diseases with unknown etiology, complement testing is recommended.

## 1 Introduction

Acute hemorrhagic leukoencephalitis (AHLE) is a rare, acute inflammatory disease with high mortality (46.5-70%), and is considered the fulminant form of acute disseminated encephalomyelitis (ADEM) (1). Presenting symptoms include rapid neurologic decline, convulsions, and fever. Tissue biopsy shows demyelination, perivascular polymorphonuclear cell accumulation, necrosis, edema, and fibrin production. Etiology and pathomechanism require further clarification. It often occurs after infections or -rarely- vaccinations, suggesting an autoimmune process driven by molecular mimicry between myelin and viral/bacterial antigens (1–3). Treatment includes high-dose methylprednisolone (HDMP), therapeutic plasma exchange (PEX), and intravenous immunoglobulin (IVIG), but decompressive craniectomy is often indicated (2).

## 2 Case report

A 9-year-old Caucasian boy presented a few days after upper respiratory illness with headache, fever, dizziness, weakness, lethargy, unsteady gait, diplopia, and blurred vision at our hospital.

At age three, he had suffered severe lobar pneumonia with pleural effusion, requiring intensive care and drainage. Blood and pleural fluid cultures had been negative.

**Figure 1:**
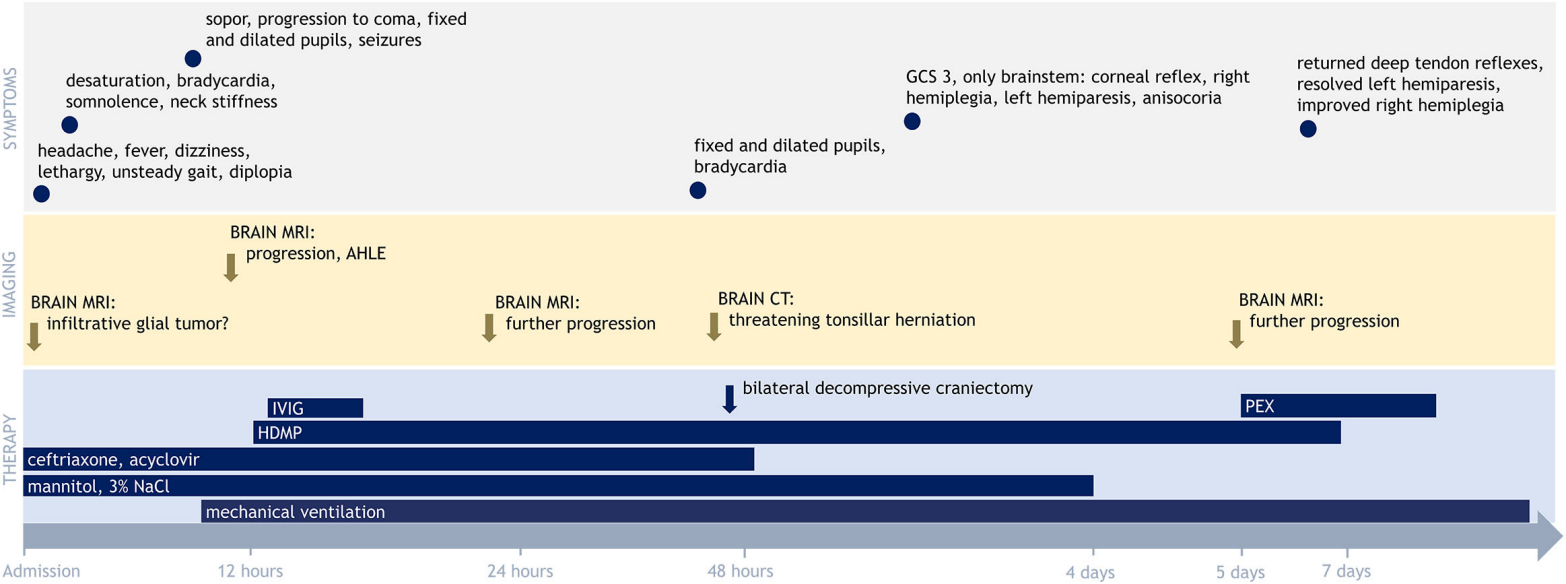
Clinical course, radiological findings, and treatment during the first 7 days after admission. AHLE, acute hemorrhagic leukoencephalitis; HDMP, high-dose methylprednisolone; IVIG, intravenous immunoglobulin; PEX, therapeutic plasma exchange; GCS, Glasgow Coma Scale. A detailed description of the clinical course, radiological findings, and treatment is provided in the main text.

**Figure 2:**
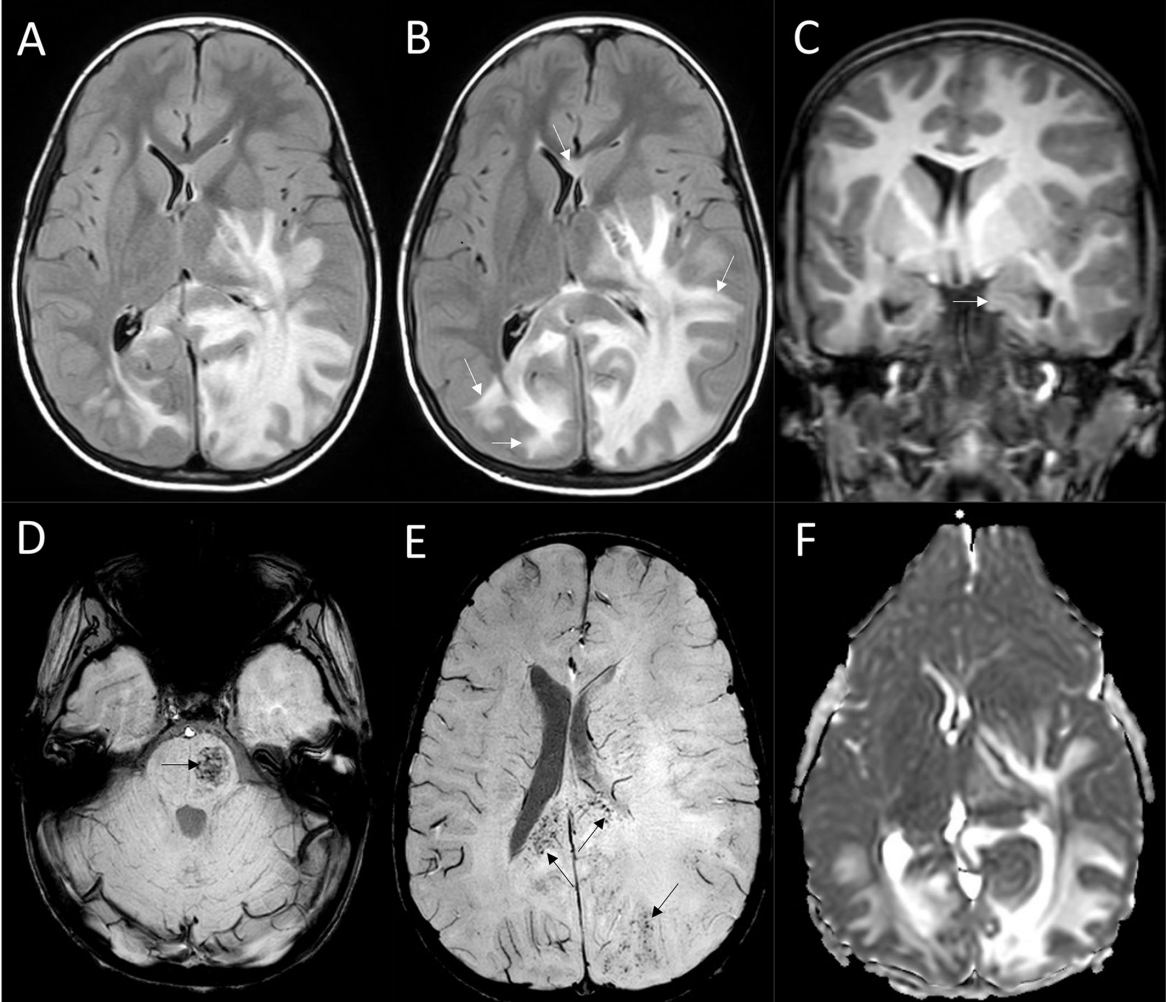
MRI findings. Axial FLAIR of the acute brain MRI **(A)** showed a confluent space-occupying lesion in the white matter of the left parietal and occipital lobes and the left thalamus and basal ganglia, sparing the cortex. The lesion extended along the corticospinal tract toward the brainstem and spread to the frontal and temporal lobes, crossing the corpus callosum to the right parietal and occipital lobes, causing a midline shift to the right. Axial FLAIR of the follow-up scan 10 hours later **(B)** presented progression (white arrows on B) and threatening uncal herniation (white arrow, **(C)**. Extensive hemorrhagic lesion developed at the left aspect of the pons (black arrow, **(D)** and the originally subtle microhemorrhages of the white matter progressed (black arrows, **(E)**. Increased diffusion indicating vasogenic oedema was demonstrated in the lesion on ADC **(F)**.

At his current admission, physical examination revealed signs of dehydration, and parenteral fluids were initiated. In the first two hours of observation, desaturation, bradycardia, somnolence and neck stiffness occurred (**Figure 1**). Empiric antimicrobial therapy with ceftriaxone and acyclovir, along with hyperosmolar therapy using mannitol and 3% NaCl, was started. Acute brain MRI showed a confluent, space-occupying lesion in the left parietal and occipital white matter with subtle microhemorrhage sparing the gray matter (**Figure 2**). The initial diagnosis was infiltrative glial tumor, but neurosurgeons did not yet recommend intervention.

Meanwhile, the patient’s neurologic status rapidly deteriorated. Gradual progression to coma developed, along with fixed, dilated pupils and purposeless movements in the upper limbs. Seizures developed, and he was treated with midazolam, but altered consciousness and intracranial pressure elevation prompted transfer to the intensive care unit for complex care and mechanical ventilation. Laboratory workup showed leukocytosis with 97% neutrophils, and an elevated C-reactive protein level (**Table 1**). CSF protein was elevated, cell count was 78 cells/µL, predominantly granulocytes (78%).

**Table 1:** Relevant laboratory results of the patient in the acute phase of the disease.

Peripheral blood			Cerebrospinal fluid		
Parameter	Value	Normal range	Parameter	Value	Normal range
CRP	69 mg/L	<10	Total cell count	78 cells/uL	–
PCT	0.55 ug/L	<0.5	WBC	78 cells/uL	–
WBC	24.15 G/L	4.5-11	Granulocytes	78%	–
Neutrophils	97%	45-70	Lymphocytes	22%	–
Eosinophils	<0.1%	<5	RBC	<1000 cells/uL	–
Basophils	<0.1%	<2	Glucose	4.8 mmol/L	2.9-4
Monocytes	0.3%	2-10	Protein	1615 mg/L	150-450

Peripheral blood			Complement system evaluation after PEX		
Parameter	Value	Normal range	Parameter	Value	Normal range
Lymphocytes	2.6%	25-45	**Complement system evaluation after PEX**		
Neutrophils	23.42 G/L	2-6.6	**Parameter**	**Value**	**Normal range**
Eosinophils	<0.01 G/L	0.03-0.5	Alternative pathway	0%	70-125
Basophils	0.02 G/L	<0.2	C3	0.79 g/L	0.9-1.8
Monocytes	0.08 G/L	0.15-0.9	C4	0.22 g/L	0.15-0.55
Lymphocytes	0.63 G/L	1.5-6.5	Factor H	338 mg/L	250-880
RBC	3.71 T/L	3.8-5	Factor I	20%	70-130
HGB	109 g/L	115-150	Factor B	20%	70-130
Hematocrit	0.3	0.36-0.47	C1q antigen	74 mg/L	60-180
MCV	80.9 fL	80-99	Anti-H factor IgG	36 AU/mL	<110
MCH	29.4 pg	27-34	Anti-C1q IgG	7 AU/mL	<52
PLT	417 G/L	150-400	SC5b-9 (terminal complement complex)	825 ng/mL	110-252

CRP, C-reactive protein; HGB, hemoglobin; MCH, mean corpuscular hemoglobin; MCV, mean corpuscular volume; PCT, procalcitonin; PEX, therapeutic plasma exchange; RBC, red blood cell; PLT, platelet count; WBC, white blood cell; AU, arbitrary units.

Ten hours after presentation repeated brain MRI revealed progression. Based on the clinical presentation and MRI findings, AHLE was suspected, and HDMP (30 mg/kg) and IVIG (2 g/kg) therapies were initiated. Follow-up brain MRI 12 hours later showed further progression in all affected regions. He had an intracranial pressure sensor placed, with an opening pressure of 20 Hgmm. A few hours later, a rapid rise in intracranial pressure to 50 Hgmm and signs of brainstem compression developed despite deepening sedation, barbiturate coma, and escalated hyperosmotic therapy. CT scan revealed threatening tonsillar herniation, requiring immediate bilateral decompressive craniectomy. Twenty-four hours after surgery, the Glasgow Coma Scale score was still 3, and the only brainstem reflex the patient had was corneal reflex. When sedation was reduced, a severe neurologic deficit became apparent: right hemiplegia, left hemiparesis, anisocoria, and absent pupillary reflexes.

Despite continued HDMP treatment, follow-up brain MRI on day five demonstrated further slow progression. We decided to escalate therapy with PEX using fresh frozen plasma. Four sessions of PEX resulted in improvement of the patient’s neurologic status: deep tendon reflexes returned, left hemiparesis resolved, and right hemiplegia improved. On day 13 accidental extubation occurred without the need for reintubation. MRI on day 20 showed significant resolution of white matter lesions. Mobilization and complex rehabilitation were initiated, fine and gross motor skills quickly improved.

Ten months after the acute episode, the patient underwent a successful cranioplasty. At 1.5 years of neurological follow-up, he exhibited no residual neurological deficits. He returned to his regular school and demonstrated academic performance comparable to his pre-illness level, maintaining his status as a high-achieving student.

Extensive etiological workup was inconclusive. CSF cultures, JC virus and West Nile virus PCRs were negative, as were serum myelin oligodendrocyte glycoprotein and aquaporin-4 antibodies, serum and CSF autoimmune encephalitis panel. The paraneoplastic panel showed nonspecific changes. Serum and CSF protein analysis revealed blood-brain barrier disruption.

**Figure 3:**
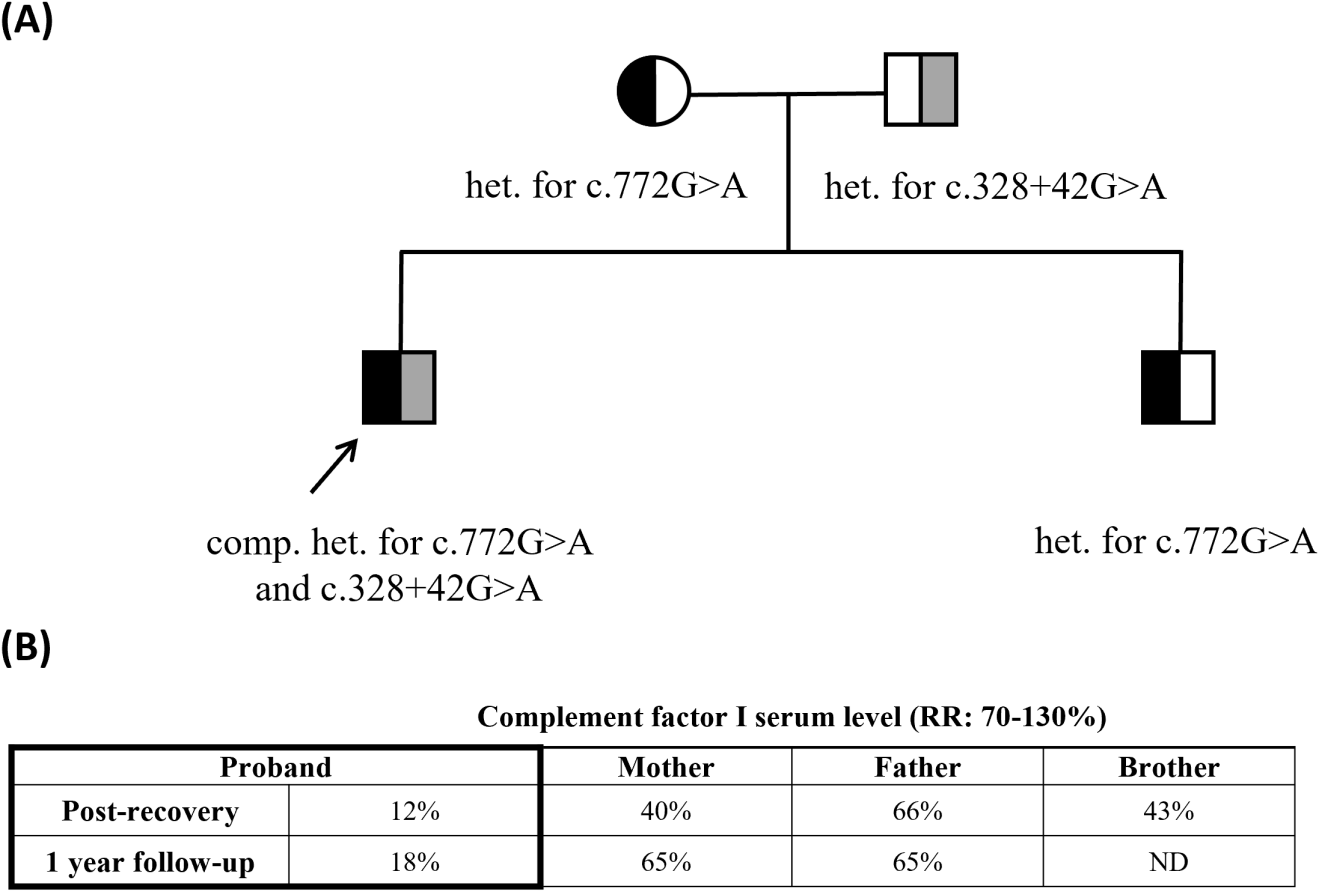
Family pedigree with identified variants and quantification of factor I serum levels over time. RR: reference range; ND: not determined; het: heterozygous; comp. het: compound heterozygous. **(A)** Family tree. Pathogenic variant (c.772G>A) maternally inherited by both siblings. Paternal intronic variant (c.328+42G>A) uniquely received by the proband. **(B)** Longitudinal quantification of complement factor I (FI) protein levels by radial immunodiffusion in family members at early clinical remission of the patient and 1-year follow-up.

Based on the history of severe pleuropneumonia and AHLE, immunodeficiency was suspected. The complement system, which is a common pathway in both infective and immune-mediated diseases, was examined through a serum complement profile applying methods published previously (4). The results showed abnormalities related to the alternative pathway and low levels of C3, factor I (FI), and factor B (FB), while C4 and C1q levels were normal (**Table 1**). The level of terminal pathway products was markedly elevated supporting severe over-activation. Repeated measurements, taken further from PEX, confirmed these results. At 12 months follow-up, the complement profile remained similar, with decreased levels of C3, FI, and FB, and slightly increased terminal pathway activation. Since this profile is characteristic of FI deficiency, the parents were also tested and both had decreased FI levels with an otherwise near-normal complement profile, raising the possibility of compound heterozygous causes in the background of FI deficiency (**Figure 3**).

Sequencing of the *CFI* gene coding region and intron-exon boundaries (as described previously (5)) revealed the heterozygous presence of a pathogenic variant affecting the last nucleotide of exon 5: NM_000204.5(CFI):c.772G>A. This previously characterized variant, causing a splicing error, is pathogenic for FI deficiency and atypical Hemolytic Uremic Syndrome (aHUS) (6). Genetic analysis confirmed the presence of the same variant in the mother and brother, but not in the father. No other pathogenic variant was detected in exons and consensus splice site regions, but a paternally derived variant was identified in the flanking region of exon 2 (c.328+42G>A). This rare base substitution potentially causes aberrant mRNA splicing and low FI protein level based on in silico prediction tools Splice AI (7) and Pangolin (8). All family members were healthy, but anamnestic data revealed abnormalities possibly related to heterozygous complement FI deficiency: recurrent childhood infections and childhood kidney failure with jaundice in the mother; recurrent infections in the adolescent sibling and retinal detachment requiring vitrectomy in the father (9).

## 3 Discussion

**Figure 4:**
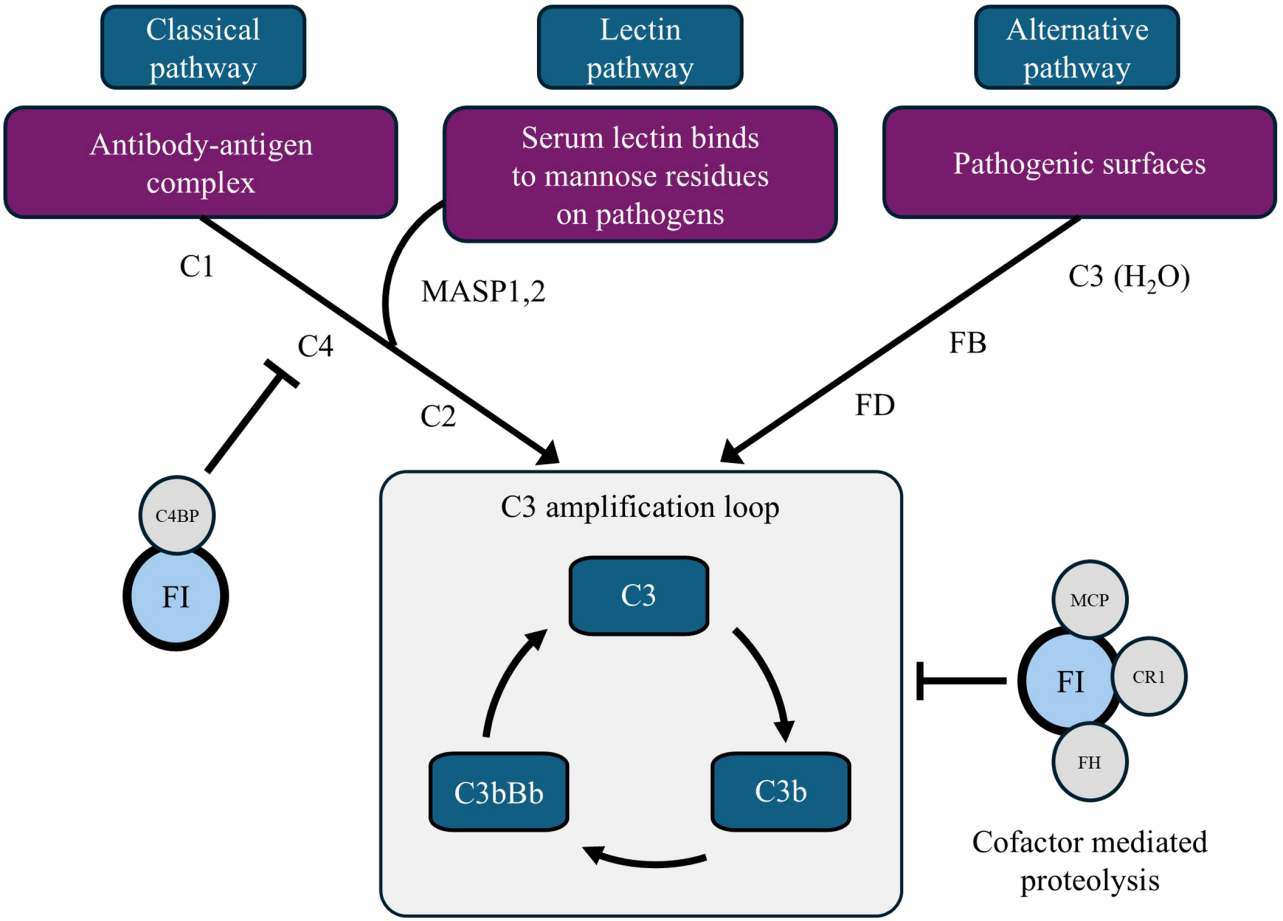
Schematic view of the role of factor I in the complement system. C4BP, C4b-binding protein; CR1, complement receptor type 1; FB, factor B; FD, factor D; FH, factor H; FI, factor I; MAC, membrane attack complex; MASP, mannose binding lectin associated serine protease; MCP, membrane cofactor protein. Complement FI is a serine protease that inactivates C3b and C4b, preventing the progression of C5 convertases in both the alternative and classical complement pathways.

Complete complement FI deficiency is a rare immunodeficiency with a limited number of reported cases in the literature (10–12). Complement FI is a serine protease that inactivates C3b and C4b, thereby preventing the conversion of C5 in both the alternative and classical pathways (**Figure 4**) (2, 13). In the absence of FI, chronic complement over-activation leads to an elevated level of the anaphylatoxin C3a, as well as a secondary consumption of C3 and FB contributing to the most typical phenotype, increased susceptibility to bacterial infections.

Besides, the diverse presentation of complete FI deficiency involves autoimmune, rheumatological, and dermatological manifestations, while a growing list of disorders is associated with incomplete FI deficiency, including aHUS and age-related macular degeneration. In recent years nine patients with both partial and complete FI deficiency were reported with neurological diseases such as AHLE, ADEM, aseptic encephalomeningitis, CNS vasculitis, and longitudinal extensive transverse myelitis (2, 11, 12, 14). The mechanism by which complete FI deficiency may lead to aseptic neuroinflammation still needs to be elucidated. The role of decreased immune-complex clearance secondary to chronic complement consumption and overproduction of complement anaphylatoxins (C3a, C5a) has been proposed, while the episodic nature may be the result of a rapid increase of the acute phase proteins, factor B and C3 in response to various stimuli (e.g. infections, menstruation) leading to the uncontrolled activation of the formerly substrate-limited alternative pathway (2, 11, 12, 14, 15). The localization of inflammation within the central nervous system may be attributed to several factors, including the low expression of complement regulators (12), the neurological mimicry or chemotactic properties of the pathogen (11), or the inherent anatomical constraints of the brain’s limited volume.

In this report we described a case with fulminant acute hemorrhagic leukoencephalitis in whom the previous upper respiratory infection may have triggered uncontrolled complement activation, leading to neuroinflammation. Immunomodulatory therapies included steroids, IVIG, and PEX. During PEX, the use of fresh frozen plasma containing exogenous FI likely facilitated the conversion of C3b to iC3b (13, 14), and possibly slowed down ongoing C3 activation. Others recommend the IL-1 antagonist anakinra and the C5 inhibitor eculizumab for the treatment of complement FI deficiency-associated AHLE (11).

Our case presented markedly reduced level of FI suggesting genetic factors in the background. Certain pathogenic and likely pathogenic *CFI* variants may result in very low or absent FI levels, while others cause normal serum FI levels with abnormal FI protein function (16). Given the observed consistently decreased FI levels in the parents and severely low levels in the proband (**Figure 3**), our patient likely inherited a low-producing allele from both parental sides. One underlying genetic cause could be unambiguously identified, as the patient inherited a well-known deficiency-causing variant (c.772G>A) from the maternal side. No pathogenic variant was identified in the coding and consensus splice sites regions from the paternal side; however, analysis of the flanking regions uncovered the presence of a rare intronic variant (c.328+42G>A) predicted to influence splicing. Functional studies, that may reveal the consequence of this variant of currently unknown significance, are ongoing. It has to be noted however, that conventional exon-directed germline sequencing mostly fails to reveal pathogenic deep-intronic variants or certain large deletions, therefore further investigation is needed to exclude the presence of such potentially pathogenic variants in the patient and his father.

Our case emphasizes that fulminant demyelinating disease can be a presenting feature of complement deficiency, underscoring the importance of considering primary immune deficiency in such cases (11). As demonstrated related to our patient, early diagnosis can have an immediate therapy-modifying impact, resulting in a significantly improved disease prognosis. In immune-mediated neurological disorders with an unknown etiology, assessment of the complement system is recommended.

### 3.1 Patient perspective

The patient made a full recovery and has remained relapse-free since. He and his parents provided informed consent to share his case for educational and scientific purposes.

## Data Availability

All relevant data is contained within the article: The original contributions presented in the study are included in the article; further inquiries can be directed to the corresponding author/s.
